# A lightweight network architecture for traffic sign recognition based on enhanced LeNet-5 network

**DOI:** 10.3389/fnins.2024.1431033

**Published:** 2024-06-18

**Authors:** Yuan An, Chunyu Yang, Shuo Zhang

**Affiliations:** ^1^China University of Mining and Technology, Engineering Research Center of Intelligent Control for Underground Space, Ministry of Education, Xuzhou, China; ^2^Xuzhou University of Technology, Jiangsu Province Key Laboratory of Intelligent Industry Control Technology, Xuzhou, China; ^3^Beijing University of technology, Faculty of Information Technology, Beijing, China

**Keywords:** traffic sign identification, automatic driving, LeNet-5, optimize activation function, convolutional neural network, space pool

## Abstract

As an important part of the unmanned driving system, the detection and recognition of traffic sign need to have the characteristics of excellent recognition accuracy, fast execution speed and easy deployment. Researchers have applied the techniques of machine learning, deep learning and image processing to traffic sign recognition successfully. Considering the hardware conditions of the terminal equipment in the unmanned driving system, in this research work, the goal was to achieve a convolutional neural network (CNN) architecture that is lightweight and easily implemented for an embedded application and with excellent recognition accuracy and execution speed. As a classical CNN architecture, LeNet-5 network model was chosen to be improved, including image preprocessing, improving spatial pool convolutional neural network, optimizing neurons, optimizing activation function, etc. The test experiment of the improved network architecture was carried out on German Traffic Sign Recognition Benchmark (GTSRB) database. The experimental results show that the improved network architecture can obtain higher recognition accuracy in a short interference time, and the algorithm loss is significantly reduced with the progress of training. At the same time, compared with other lightweight network models, this network architecture gives a good recognition result, with a recognition accuracy of 97.53%. The network structure is simple, the algorithm complexity is low, and it is suitable for all kinds of terminal equipment, which can have a wider application in unmanned driving system.

## 1 Introduction

With the rapid development of social economy and the arrival of the era of artificial intelligence, information and intelligence have become the focus of social attention, and automatic driving has become one of the current hot research fields. Automatic driving system is a comprehensive system integrating information detection, information communication and intelligent control technology (Chen et al., 2018; Zhang et al., 2019). It can realize the interaction and coordination among people, vehicles and roads, so as to effectively improve road traffic conditions and travel efficiency. The international automotive industry has recognized two categories of autonomous driving classification standards: National Highway Traffic Safety Administration and the Institute of Automotive Engineers, of which the second classification standard is more widely used (Hur and Kang, 2020).

Automatic driving in the world is in the stage of partial automation. Even if it makes great progress in the laboratory, it still faces many difficulties and challenges, and there is still a long way to go before entering the market commercially. In automatic driving, traffic sign recognition is an important part. The traffic signs on the road are rich in information, including very important traffic information, which can provide necessary road guidance for intelligent driving. Accurately identifying traffic signs and issuing correct driving instructions can effectively reduce the possibility of traffic accidents and improve driving efficiency. Therefore, the research of traffic sign recognition has important research significance and practical value, and has gradually become a hot research topic in related fields. As an important part of the automatic driving system, the traffic sign detection system mainly uses the vehicle-mounted camera to shoot the scene of the traffic road to obtain the required data set information (Cuesta-Infante et al., 2020). The comprehensive application of computer image processing technology, artificial intelligence technology and big data technology to detect and identify traffic signs can provide effective traffic information for the control platform of automatic driving, so as to increase the reaction time of the automatic driving control system and improve the safety of automatic driving. However, there are many factors affecting traffic sign recognition on the actual road: complex backgrounds, light and dark weather, weather factors, occlusion damage, signs aging, fading, etc. These factors bring great challenges to traffic sign detection. At the same time, the performance of automatic driving terminal equipment is quite different, which puts forward higher requirements for the complexity of traffic sign detection algorithm. The network architecture of traffic sign recognition algorithm needs to meet the requirements of lightweight to adapt to the low-end automatic driving terminal equipment. Therefore, it is of great significance to design a lightweight traffic sign recognition network architecture with high precision, good real-time performance and convenient terminal deployment for the advancement of autonomous driving. When the vehicle is running at high speed on the road, the lightweight traffic sign recognition network can help the control platform to recognize the traffic sign in time. At the same time, the lightweight network architecture has low requirements on the memory and configuration of the terminal equipment. Therefore, the study of lightweight traffic sign recognition network in complex environment is still a key problem in the field of control.

## 2 Related work

At present, traffic sign detection methods mainly include template matching method, traditional machine learning method and deep learning method. The traffic sign detection method based on template matching uses the unique shape of traffic signs to match the features of the template. According to different signs indicating functions, each traffic sign has a special color and shape. Traditional recognition methods based on color and shape are widely used. In the literature (Lasota and Skoczylas, 2016; Sheikh et al., 2016; Huang and Hou, 2017; Song et al., 2017; Jain and Gianchandani, 2018; Rahmad et al., 2018) many color segmentation methods are used to implement the algorithm. The common point of this method is to use color threshold segmentation to obtain traffic signs in images. There are also other studies based on shape methods that are extensively utilized in traffic sign recognition, such as Hough transform (Moon and Lee, 2015; Onat and Ozdil, 2016; Sun et al., 2019) and angle detection (Lin and Sie, 2019; Flores-Calero et al., 2020; Ozturk et al., 2020). The generalized Hough transform has many applications, most of which are used to identify standard geometric shapes, such as circles, triangles, and rectangles. In addition, in terms of positioning symbols, the method of using color segmentation for rough estimation (Yakimov and Fursov, 2015; Filatov et al., 2017; Lee and Kim, 2018) is also commonly used, and the target is determined through preliminary information screening. Color segmentation and shape-based methods have a common feature, that is, they are sensitive to shadows, extreme weather conditions, crowded scenes and other external factors. This kind of algorithm has strict requirements on the characteristics of traffic signs, and it can only effectively detect a certain kind of traffic signs that match well with the template. This kind of algorithm has poor robustness and is more sensitive to the change of environmental factors. In the case of deformation or pollution of traffic signs, the detection accuracy drops sharply.

Based on the traditional machine learning method, by analyzing the characteristics of different traffic signs, the corresponding classifier is selected to classify the traffic signs. Wu et al. (2020) used the histogram of oriented gradient (HOG) feature to extract the edge information of the image and the local binary pattern (LBP) feature to extract the internal texture information of the image, then they fused the two extracted information with features, finally they used the extreme learning machine (ELM) classifier to classify traffic signs and tested them on the GTSRB data set, and the recognition accuracy was 92.88%. Ruta et al. (2010) used HOG features to extract features of traffic signs in images and input them into the support vector machine (SVM) classifier for training and classification. This method was tested on GTSRB data set, and the recognition accuracy was 95.68%. The detection accuracy of this method is greatly improved. However, due to the large amount of redundant information generated in the process of acquiring the region of interest, the detection speed of the algorithm is reduced. As a result, most of these algorithms cannot meet the real-time requirements of the actual scene.

Convolutional neural network is a kind of deep learning network widely used in the field of machine vision. Different from the traditional artificial neural network structure, it contains very special convolutional layer and pooling layer, which are combined through local connection and weight sharing, and use the back propagation algorithm to adjust the weight and bias adaptively. Therefore, it no longer relies too much on the prior knowledge and manual intervention of technical experts and scholars.

The traffic sign detection method based on convolutional neural network utilizes multi-layer deep learning network to independently learn and extract different features of traffic signs. This method can effectively reduce the subjective singleness of traditional methods in image feature extraction, and can solve the problem of insufficient semantic information of traditional methods in feature extraction, and has been widely used in the field of intelligent transportation.

Krizhevsky et al. (2017) proposed the use of convolutional neural networks to identify targets, thus enabling the rapid development and application of convolutional neural networks in the field of target detection. In recent years, with the rapid development of deep learning (Silver et al., 2016, 2017; Moravík et al., 2017), The neural network model based on deep learning has received much attention because of its ability to capture the dynamic characteristics of traffic sign image data and obtain the best recognition effect. Traffic sign recognition methods based on various deep convolutional neural networks have achieved some results. In the literature (Qian et al., 2015), researchers proposed a method of traffic sign detection and recognition. This method uses the features of the multi-task convolutional neural network for training, so as to obtain the geographical indications of various traffic signs, and effectively determine the classification features. Some scholars (Zhu et al., 2018) use a fast neural network to extract the candidate regions of interest provided by the previous complete convolutional network, and then determine the target value through text detection. Literature (Yao et al., 2017) proposed an field-programmable gate array (FPGA)-based convolutional neural network module with better automatic recognition performance. Compared with the traditional convolutional neural network model, its performance on the hardware platform is better. The energy consumption in traffic sign recognition is smaller, and the accuracy is higher, but there are certain support requirements for the hardware platform. There are also some studies that have made some progress in semantic segmentation based on deep convolutional neural networks (Zhao et al., 2017) and object recognition (Liang and Zhang, 2015). In some specific complex scenes, convolutional neural networks can use high-level semantic information as a feature method to solve some tasks (such as occlusion and various targets). In the traditional traffic sign recognition method, the color and shape information are limited and the high-level semantics to define the direction of the target space are lacking, so it cannot meet the higher-level needs. In the literature (Zang et al., 2016), scholars use cascaded convolutional neural networks to recognize traffic signs and operate on the previously extracted selected regions, thus showing good performance. However, its application platform is relatively limited, and it is not an end-to-end traffic sign recognition. Hussain et al. (2018) proposed a CNN fast branching model, which highly mimics biological mechanisms to improve efficiency. In terms of accuracy, the performance is acceptable, and the potential application possibilities are greater, but the efficiency under time-sensitive conditions is worth exploring. Mehta et al. (2019) proposed a method for the classification of traffic signs based on deep convolutional networks. Good optimization results can be obtained through Adam optimizer, and softmax activation also has certain performance. However, the classification accuracy needs to be improved. In addition, some scholars have proposed convolutional neural network algorithms based on driving and multi-column types (Karaduman and Eren, 2017; Shi and Yu, 2018). These classification networks are getting deeper and deeper, their structure is becoming more and more perfect, and the effect is getting better and better.

With the gradual increase of algorithm complexity, the accuracy of traffic sign recognition has been greatly improved, but at the same time, the application of terminal setting is gradually limited. Although large-scale convolutional neural network models have superior performance, they also bring the problems of high memory consumption. How to apply convolutional neural network to unmanned mobile devices requires directly facing the two major problems of storage and speed. The lightweight model is concerned with designing more efficient network computing methods so as to reduce network parameters without losing too much accuracy. Therefore, how to design a lightweight high-performance target detection has quite high application value and scientific research significance. LeNet-5 is one of the classical convolutional neural networks, and has been widely used in the field of image recognition since it was proposed. Based on LeNet-5, this paper proposes a lightweight traffic sign recognition network that meets the requirement of intervention time, providing a reference for terminal network deployment.

## 3 Methods

### 3.1 Image data processing

Traffic sign image recognition is a challenging problem. There are many differences in color, shape and hieroglyphics of traffic signs, which makes the image recognition of traffic signs become an unbalanced multi-class recognition problem. Although some commercial recognition systems have been put into the market and many related research reports have been published, before the advent of the GTSRB dataset, there is still a lack of benchmark data to fairly evaluate different image recognition methods. The GTSRB dataset is a multi-class benchmark dataset for image classification, and the application algorithm needs to recognize a single image of traffic sign. The images in the GTSRB dataset come from images or videos obtained from onboard cameras, and each type of traffic sign appears only once (Stallkamp et al., 2012). The data set contains 43 traffic sign categories, totaling more than 50,000 images (Saadna and Behloul, 2017). Each traffic sign type contains between 210 and 2,250 images to train and test the algorithmic model’s ability to recognize various types of traffic signs.

The training folder of the GTSRB dataset contains 39,209 images, and we used the remaining 12,630 images as the test set. For the training set and the verification set, we divided the images in the training folder according to the ratio of 8:2, that is, the training set was 31,433 images, and the verification set was 7,776 images. The annotation tags are stored in a *csv* file, including location tags and 43 types of traffic light tags, which are cropped and scaled to a fixed size by the location tags. The data format is shown in Table 1.

**TABLE 1:** Data format.

Data set	Number of pictures	Number of annotation images	Number of sign types	Size of signs	Source of pictures	Acquisition mechanism
GTSRB	133000–144769	51840	43	15 × 15–250 × 250	Germany	Prosilica GC 1380ch color camera

Since the data set is collected in the real environment, the training data set is very unbalanced due to weather conditions, light changes, occlusion, motion and other problems, and the image has changes such as blur, distortion, rotation, etc., so it is necessary to preprocess the data set to enhance the robustness of the model.

#### 3.1.1 Image resizing

The image aspect ratio in the dataset used in this study ranges from 15 × 15 to 250 × 250 pixels. In order to be compatible with neural networks, there must be a fixed image size (Sultana et al., 2020). It is worth noting that reducing the image size to a lower pixel value reduces the complexity of the model, but may also negatively affect the accuracy of the model and may reduce the classification performance of the algorithm. During the experiment, we tested our model with different image pixel sizes and found that 32 × 32 pixels provided the best trade-off between computational complexity and classification accuracy. So we resize the image to 32 × 32.

#### 3.1.2 Color conversion

The image color information is more sensitive to the lighting conditions and the quality of the capturing equipment (Sudharshan and Raj, 2018). Meanwhile, the number of training parameters and training time of gray-scale images are reduced compared with color images (Bui et al., 2016; Sudharshan and Raj, 2018). In the process of data processing, considering that color is not the main feature of traffic sign recognition, the color image is converted into a grayscale image, and the single-channel image is iterated more quickly. The weighted average method is used to process the grayscale of the picture, and the three RGB components representing the picture are weighted and averaged with different weights according to their importance and other indicators. Considering the difference in sensitivity of the human eye to green and blue, the weighted average of the three components of RGB can obtain a more reasonable gray-scale image, as shown in formula (1).


(1) F⁢(i,j)=0.30⁢R⁢(i,j)+0.59⁢G⁢(i,j)+0.11⁢B⁢(i,j)

#### 3.1.3 Histogram equalization

Histogram equalization is a nonlinear stretching operation to redistribute image pixel values so that the number of pixels in a certain gray range is roughly the same. Histogram equalization helps to equalize image brightness distribution, which can increase image contrast and make image details clearer (Dhal et al., 2021). The image histogram equalization method is shown in formula (2).


(2) $$S_{k}=T\,\left(r_{k}\right)=\sum_{j=0}^{k}P_{r}\,\left(r_{j}\right)=\sum_{j=0}^{k}\frac{n_{j}}{n}$$

Where, *r_k_* is the gray level contained in the image, *n_k_* represents the number of the k*th* gray level, *S_k_* is the gray level output after calculating the mapping using the transformation function.

**FIGURE 1:**
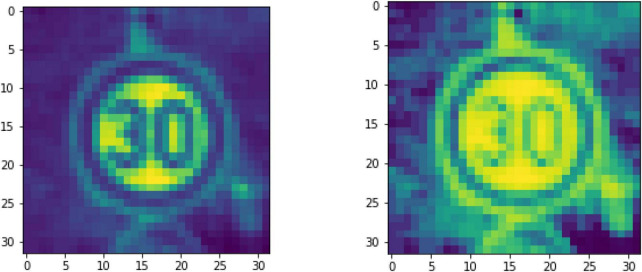
Comparison before and after histogram equalization.

The picture is scaled to a size of 32 × 32, and then the color image is converted to grayscale, the image to be displayed is randomly selected, and the histogram of the balanced picture is obtained according to (1), as shown in Figure 1.

In Figure 1, it should be noticed that the download data set format provided by GTSRB is in *ppm* format, which needs to be converted to *jpg* format. The images in the data set have surrounding backgrounds. According to the cropped area, it is scaled to a “fixed size.” Here it can be realized by MATLAB script, including how to read the label information of the image in *csv* and how to convert the *ppm* format to *jpg* format, and finally preprocess the obtained pictures.

#### 3.1.4 Data normalization

Data normalization is essential to ensure a uniform distribution of input parameters (pixel values), which allows the network architecture to converge quickly during training. Standardize the input image and process the input features into a similar range, thus making the optimization of the cost function easier and faster (Akshata and Panda, 2019; Zaibi et al., 2021). In this project, the training set and test set are normalized to the range (−1, 1). The min-max standardization method is used to normalize the data. However, the min-max standardization method is to transform the original data into the [0, 1] interval, so we first make improvements, as shown in Formula (3).


(3) $$x^{*}=\frac{x-x\,\_\,a\,v\,e\,r\,g\,e}{x_{m\,a\,x}-x_{m\,i\,n}}$$

As shown in formula (3), the data is processed, considering that in order to avoid recalculating the value each time, we set *x*___*averge* to a fixed value of 128. The normalized processing results are shown in Table 2.

**TABLE 2:** Normalization result.

i	array[i]	i	array[i]	i	array[i]	i	array[i]
1	-0.859375	9	-0.625	17	0.1171875	25	0.0078125
2	0.9609375	10	-0.625	18	0.171875	26	0.125
3	0.9765625	11	-0.375	19	0.3359375	27	0.1171875
4	0.9296875	12	-0.375	20	0.171875	28	-0.1875
5	-0.859375	13	0.2890625	21	0.0546875	29	-0.1875
6	-0.859375	14	0.171875	22	0.125	30	0.0625
7	-0.625	15	0.6171875	23	0.125	31	0.125
8	-0.484375	16	0.3359375	24	0.0078125	32	0.171875

#### 3.1.5 Data enhancement

Data enhancement is an important step to solve the problem of unbalance of data sets. Various kinds of transformation processing are carried out on existing data to generate new data to expand the amount of data (Patil et al., 2021; Singh and Malik, 2022). Translation, scaling and rotation are commonly used transformation means. At the same time, it can ensure that there are no identical traffic sign images in the data set, so the robustness of the model is improved.

In the process of traffic sign recognition training, the original data is trained, and the results show that the accuracy of the training set is high, while the accuracy of the verification set is low, which is manifested as overfitting. When over-fitting occurs, the high accuracy of the training set indicates that the algorithm has fully learned the features of the original data, while the accuracy of the validation set is low, indicating that the characteristics of the original data are insufficient, which makes the algorithm in the new validation set the performance is poor (Akshata and Panda, 2019; Radu et al., 2020; Patil et al., 2021). In the actual scene, the angle of the traffic sign changes. In this case, model training will make the network convergence speed relatively slow, and the model effect is relatively poor. This paper adopts Keras-image-data-augmentation, a lightweight library based on Keras, for Image Data enhancement. We can set rotation Angle, translation distance, scaling ratio, etc., to simulate images under different perspectives. At the same time, in the training process, each iteration of the system will produce a new, randomly transformed image, which can avoid overfitting the model to a specific data pattern.

### 3.2 Model structure

LeNet-5 is one of the classical convolutional neural networks. LeNet-5 model has a good performance in the field of digit symbol recognition, and it is also helpful for the development of traffic sign recognition. However, due to its limitations, it usually performs poorly. Therefore, the LeNet-5 network model is improved in this paper to achieve better performance in symbol recognition scenarios. The model originally used the S-type activation function, but as the value tends to infinity, the images on the left and right sides of the function tend to be flat, and the gradient value gradually tends to 0, which can easily cause the gradient to disappear and slow down the convergence speed of the model. In order to improve the sigmoid function, this inherent limitation causes the accuracy of the model to decrease. In terms of the pool layer, the LeNet-5 network model originally used the average pool, but the average pool averaged the values within the filter range to obtain the output. In the field of image recognition, max pooling is used more frequently, and the maximum value in the filter range is used as the output, which has strong robustness.

**FIGURE 2:**
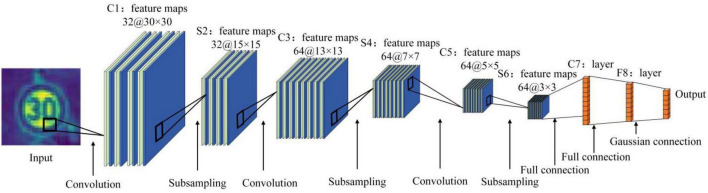
Adaptive network structure diagram.

The adaptive convolutional neural network model consists of an input layer, an output layer, three convolutional layers (C1, C3, C5), three pooling layers (S2, S4, S6) and a fully connected layer (F7). Input layer: The input is a sample image with a size of 32 × 32 pixels. Convolutional layer C1: the input layer is convolved using thirty-two convolution cores of size 3 × 3 with a step size of 1. A convolution kernel will obtain a feature map, so this layer consists of thirty-two feature maps. Pooling layer S2: S2 adopts a maximum pooling strategy, which is obtained after down-sampling at the C1 layer. The size of the pooling area in S2 is 2 × 2, and the step size is 1. Convolutional layer C3: sixty-four convolution cores of size 3 × 3 with a step size of 1. Each feature map in C3 is a weighted combination of all thirty-two or more feature maps in S2. The output is sixty-four 13 × 13 feature maps. Pooling layer S4: S2 adopts a maximum pooling strategy, which is obtained after down-sampling at the C1 layer. The size of the pooling area in S2 is 2 × 2, and the step size is 1. Convolutional layer C5: sixty-four convolution cores of size 3 × 3 with a step size of 1. A convolution kernel will obtain a feature map, so this layer is composed of sixty-four feature maps. Pooling layer S6: S2 adopts a maximum pooling strategy, which is obtained after down-sampling at the C1 layer. The size of the pooling area in S2 is 2 × 2, and the step size is 1. Output layer: fully connected layer, the logo corresponds to the output image. The adaptive network structure is shown in Figure 2.

As shown in Figure 2, the reconstruction of the pooling layer is conducive to the rapid recognition of traffic signs in the intelligent driving environment, and at the same time can improve the accuracy. The model structure table is shown in Table 3.

**TABLE 3:** Model structure.

Layer (type)	Output shape	Param
conv2d (Conv2D)	(None, 30, 30, 32)	320
p_re_lu (PReLU)	(None, 30, 30, 32)	28800
max_pooling2d (MaxPooling2D)	(None, 15, 15, 32)	0
dropout (Dropout)	(None, 15, 15, 32)	0
conv2d_1 (Conv2D)	(None, 13, 13, 64)	18496
p_re_lu_1 (PReLU)	(None, 13, 13, 64)	10816
max_pooling2d_1 (MaxPooling2D)	(None, 7, 7, 64)	0
conv2d_2 (Conv2D)	(None, 5, 5, 64)	36928
p_re_lu_2 (PReLU)	(None, 5, 5, 64)	1600
max_pooling2d_1 (MaxPooling2D)	(None, 3, 3, 64)	0
flatten (Flatten)	(None, 576)	0
dense (Dense)	(None, 512)	295424
dropout_1 (Dropout)	(None, 512)	0
dense_1 (Dense)	(None, 43)	22059

### 3.3 Activation function

Activation function is a nonlinear mapping of the predicted results, which can improve the resolution of the model, so that the model has the ability to deal with complex problems and high learning ability. In convolutional neural networks, activation functions including Sigmoid, Tanh, ReLU, Leaky-Relu, ELU, and Maxout are frequently utilized.

Through the analysis of the activation function, the Sigmoid function and the Tanh function are saturated nonlinear functions, and the convergence speed is slow, which is easy to cause gradient explosion or gradient small phenomenon. The ReLU function does not have the problem of gradient disappearance, and there will be no saturation problem. Therefore, ReLU can maintain the gradient without attenuation, which alleviates the problem of gradient disappearing, so that we can directly train deep learning neural networks in a supervised manner, without relying on unsupervised layer-by-layer pre-training. However, with the development of training, the “vulnerability” of the ReLU function has gradually become apparent. At that time, the derivative of the function is always 0, which prevents false responses.

The Leaky-ReLU function is an improvement of the ReLU function when the gradient is a range index. In order to solve the problem of regional neuron disappearance, the Leaky-ReLU function only replaces horizontal lines with non-horizontal lines. In the specific direction propagation process of the model, input the Leaky-ReLU activation function, the part less than zero can prevent the neurons in the area from becoming dead neurons, and the gradient can also be calculated. However, according to different values, the role of the Leaky-ReLU function is also different, and its function is shown in formula (4).


(4) $$f\,\left(x\right)=\left\{ \begin{array}{cc} x_{i}, & x\geq 0\\ \alpha_{i}\,x_{i}, & x<0 \end{array}\right.$$

As shown in formula (4), the PReLU function is an improvement of the Leaky-ReLU function. In the PReLU function, α is a trainable function, and the neural network will also learn the value of α to achieve faster and better convergence. The nonlinear activation input on the channel is a coefficient that controls the slope of the negative part, which allows the nonlinear activation function to have different values on different channels, and the PReLU degenerates to ReLU. When the value is small and fixed, PReLU will degenerate into LReLU, and PReLU will only increase a very small number of parameters. Compared with the total number of parameters, these additional parameters can be ignored, so this also means that the risk of overfitting will only increase a little. Especially when different channels use the same α, there are fewer parameters. PReLU can be trained in the back propagation process at the same time, and can be optimized together with other layers. The update formula is derived from the chain rule, and the gradient of each layer is shown in formula (5).


(5) $$\frac{\partial \varepsilon }{\partial \alpha_{i}}=\sum_{y_{i}}\frac{\partial \varepsilon }{\partial f\,\left(y_{i}\right)}\,\frac{\partial f\,\left(y_{i}\right)}{\partial \alpha_{i}}$$

As shown in formula (5), εrepresents the objective function, $\frac{\partial \varepsilon }{\partial f\,\left(y_{i}\right)}$ is the gradient propagated from a deeper layer, and its activation gradient is shown in formula (6).


(6) $$\frac{\partial f\,\left(y_{i}\right)}{\partial \alpha_{i}}=\left\{ \begin{array}{c} 0,y_{i}>0\\ y_{i},y_{i}\leq 0 \end{array}\right.$$

Acting on all element maps, for channel shared variables, the gradient is shown in formula (7).


(7) $$\frac{\partial \varepsilon }{\partial \alpha }=\sum_{i}\sum_{y_{i}}\frac{\partial \varepsilon }{\partial f\,\left(y_{i}\right)}\,\frac{\partial f\,\left(y_{i}\right)}{\partial \alpha_{i}}$$

As shown in formula (7), ∑*y*_*i*_ is the total value of all channels. When the model updates α, the update method using the time driving quantity is shown in formula (8).


(8) $$\Delta \,\alpha_{i}:=\mu \,\Delta \,\alpha_{i}+\delta \,\frac{\partial \varepsilon }{\partial \alpha_{i}}$$

As shown in formula (8), μ represents momentum and δ is the learning rate.

## 4 Evaluation of result

In order to verify the superiority of the improvement of the PReLU activation function, by using the GTSRB dataset, the PReLU improvement function is compared with the commonly used activation functions such as Sigmoid, Tanh, SELU, ReLU, and Leaky-ReLU. We use different activation functions in the model and compare them during the 100% training process to ensure the fairness and rationality of the results. We have selected the training progress after 0%, 20%, 50%, 75%, and 100%, and their corresponding loss index and precision index are compared. As the training progress increases, the loss and accuracy of each functional model are shown in Tables 4–9.

**TABLE 4:** Comparison of each function loss and accuracy when training progress is 0%.

		Function					
Index		Sigmoid	Tanh	SELU	ReLU	Leaky-ReLU	PReLU
Train	Loss	3.6711	0.8787	0.7477	1.1660	1.1496	1.7159
	Accuracy	0.0498	0.7611	0.7914	0.6744	0.6803	0.5110
Test	val_loss	3.1683	0.5052	0.5829	0.6142	0.7049	0.7237
	val_accuracy	0.1930	0.8358	0.8308	0.8054	0.7755	0.7773

**TABLE 5:** Comparison of each function loss and accuracy when training progress is 25%.

		Function					
Index		Sigmoid	Tanh	SELU	ReLU	Leaky-ReLU	PReLU
Train	Loss	0.0094	5.42e-05	1.3126e-05	0.0016	0.0090	0.0126
	Accuracy	0.9981	1.0000	1.0000	0.9995	0.9973	0.9954
Test	val_loss	0.5266	0.2732	0.3844	0.3384	0.3882	0.1504
	val_accuracy	0.8778	0.9413	0.9268	0.9390	0.9315	0.9633

**TABLE 6:** Comparison of each function loss and accuracy when training progress is 50%.

		Function					
Index		Sigmoid	Tanh	SELU	ReLU	Leaky-RELU	PReLU
Train	Loss	1.6920e-04	4.1051e-06	9.4398e-07	1.111e-06	1.2186e-06	0.0078
	Accuracy	1.0000	1.0000	1.0000	1.0000	1.0000	0.9977
Test	val_loss	0.6174	0.3036	0.4442	0.3687	0.4419	0.1906
	val_accuracy	0.8918	0.9433	0.9293	0.9449	0.9420	0.9653

**TABLE 7:** Comparison of each function loss and accuracy when training progress is 75%.

		Function					
Index		Sigmoid	Tanh	SELU	ReLU	Leaky-ReLU	PReLU
Train	Loss	4.6383e-05	2.9061e-07	6.6417e-08	9.264e-08	9.1472e-08	0.0062
	Accuracy	1.0000	1.0000	1.0000	1.0000	1.0000	0.9982
Test	val_loss	0.6762	0.3513	0.5186	0.4361	0.5251	0.1598
	val_accuracy	0.8902	0.9424	0.9286	0.9454	0.9426	0.9732

**TABLE 8:** Comparison of each function loss and accuracy when training progress is 100%.

		Function					
Index		Sigmoid	Tanh	SELU	ReLU	Leaky-ReLU	PReLU
Train	Loss	6.5922e-06	3.0431e-08	6.1023e-09	8.242e-09	7.6751e-09	0.0052
	Accuracy	1.0000	1.0000	1.0000	1.0000	1.0000	0.9986
Test	val_loss	0.7304	0.3728	0.5822	0.4892	0.5959	0.2095
	val_accuracy	0.8943	0.9444	0.9295	0.9449	0.9415	0.9753

**TABLE 9:** Comparison of results on the test set before and after training.

	Function					
Index	Sigmoid	Tanh	SELU	ReLU	Leaky-ReLU	PReLU
Before training	0.1930	0.8358	0.8308	0.8054	0.7755	0.7773
After training	0.8902	0.9444	0.9295	0.9449	0.9415	0.9753

It can be seen from Table 4 that when the training progress is 0%, the performance of the improvement function of PReLU is not particularly ideal, and the performance on the test set is only better than the activation functions of Sigmoid and Leaky-ReLU. The results of training progress of 25% are shown in Table 5.

In the intermediate stage of the training progress, we choose the intermediate state of 50% to view the performance of each activation function, as shown in Table 6.

It is not difficult to see from Table 6 that when the training progress reaches 50%, the accuracy of other activation functions on the training set even reached 100%. Similarly, the result of PReLU on the training set also reached 0.9977, but the accuracy of the other activation functions on the test set is still slightly lower than the accuracy of the PReLU activation function (0.9653). Select 75% progress status to view the performance of each activation function, as shown in Table 7.

Select 100% progress status to view the performance of each activation function, as shown in Table 8.

In order to verify the role of the activation function, and also to compare the performance of each activation function, we compare the state before and after training, and the results are shown in Table 9 below.

**FIGURE 3:**
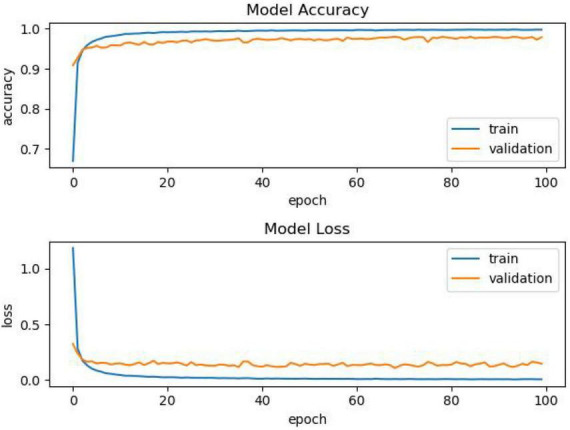
Multiple comparison char.

In Table 9, after 100% training, the performance of the PReLU improvement function is significantly better than other activation functions, verifying the effectiveness of the model. It can be seen from the above table comparison that when the model is not trained, that is, when the training progress is 0%, the effects of the activation functions of Tanh, SELU, and ReLU are better than those of PReLU improvement. However, as the training progress increases, the accuracy of the PReLU improvement function gradually increases. When the training progress is only 25%, it can be seen that the accuracy of the PReLU improvement function exceeds that of other activation functions under normal conditions. In order to compare PReLU with other activation functions, we select the model of one of the functions, and filter the images whose accuracy and loss change as the number of epochs increases, as shown in Figure 3.

**FIGURE 4:**
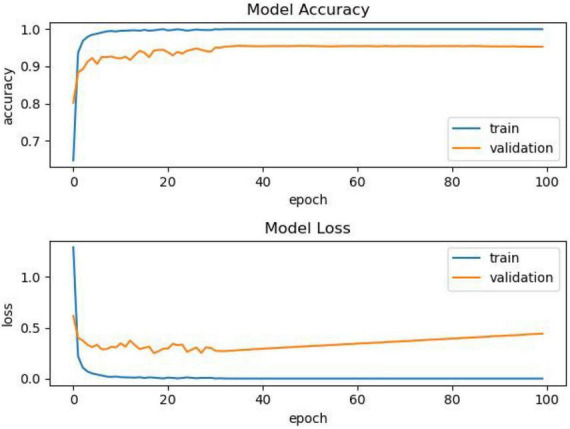
Comparison chart.

It can be seen from Figure 4 that as the training progress increases, the model using other activation functions has a relatively stable performance, but after the training, we can see that the accuracy is insufficient. The comparison of another model is shown in Figure 4.

As shown in Figure 4, as the number of epochs increases, the accuracy of using the PReLU activation function model continues to improve, and the overall effect is better than that of models using other activation functions.

In order to verify the recognition performance of the optimized LeNet-5 network, we compared the recognition accuracy with other traffic sign recognition methods on the GTSRB data set, and the comparison results are shown in Table 10.

**TABLE 10:** Comparison of recognition performance of different algorithms on GTSRB data sets.

Algorithm	Recognition accuracy
Fast R-CNN	90.1%
Faster R-CNN	91.8%
Traditional LeNet-5 network (Zhang et al., 2021)	95.48%
Optimized LeNet-5 network	97.53%

It can be seen from Table 10, compared with three typical deep learning networks (Fast R-CNN, Faster R-CNN and traditional LeNet-5 network), the optimized LeNet-5 network can extract more effective features from the dataset, and the identification accuracy is significantly improved, which can meet the requirements of automatic driving. At the same time, compared with the large complex network structure, the optimized network proposed in this paper reduces the number of neurons through weight sharing and local receptive field, achieving the purpose of reducing training parameters and improving training speed, thus greatly shortening the time of feature extraction and recognition, and making it possible to recognize traffic signs in real-time monitoring. The optimized network proposed in this paper reflects the good performance of the network model because of its simple structure and lower requirements for terminal equipment, and can be well applied in the field of traffic sign recognition.

## 5 Conclusion

Aiming at the requirements of intelligent driving for traffic sign recognition, a light traffic sign recognition network based on neural network is proposed. By improving the spatial pool convolutional neural network, the neuron nodes are optimized, and the normalized image is preprocessed. And activation, optimize the activation function to improve the recognition effect. The experimental results show that this method has a high recognition rate, especially after the improvement of the activation function, the recognition accuracy has also improved. Due to its simple structure and low requirements on terminal device memory and configuration, the network model can be effectively applied to intelligent driving scenarios. At the same time, it has a reliable recognition effect in traffic sign recognition, improving the quality and safety of intelligent driving.

## Data availability statement

The original contributions presented in this study are included in this article/supplementary material, further inquiries can be directed to the corresponding author.

## Author contributions

YA: Writing – original draft. CY: Writing – review & editing. SZ: Writing – original draft.
